# Cost-effectiveness of left atrial appendage closure for stroke prevention in atrial fibrillation: a systematic review appraising the methodological quality

**DOI:** 10.1186/s12962-023-00486-0

**Published:** 2023-10-23

**Authors:** Sumudu A. Hewage, Rini Noviyani, David Brain, Pakhi Sharma, William Parsonage, Steven M. McPhail, Adrian Barnett, Sanjeewa Kularatna

**Affiliations:** 1 Australian Centre for Health Services Innovation and Centre for Healthcare Transformation, Queensland University of Technology, No.61, Musk Avenue, Kelvin Grove, QLD 4059 Australia; 2 Department of Pharmacy, Udayana University, Bali, Indonesia; 3 Cardiology department, Royal Brisbane and Women’s Hospital, Herston, QLD Australia; 4 Digital Health and Informatics Directorate, Metro South Health, Brisbane, QLD Australia

**Keywords:** Methodological quality, Cost-effectiveness, Left atrial appendage closure, Left atrial appendage occlusion, Oral anticoagulants, Novel oral anticoagulants, Warfarin, Stroke prevention, Atrial fibrillation

## Abstract

**Background:**

The increasing global prevalence of atrial fibrillation (AF) has led to a growing demand for stroke prevention strategies, resulting in higher healthcare costs. High-quality economic evaluations of stroke prevention strategies can play a crucial role in maximising efficient allocation of resources. In this systematic review, we assessed the methodological quality of such economic evaluations.

**Methods:**

We searched electronic databases of PubMed, EMBASE, CINAHL, Cochrane Central Register of Controlled Trials, and Econ Lit to identify model-based economic evaluations comparing the left atrial appendage closure procedure (LAAC) and oral anticoagulants published in English since 2000. Data on study characteristics, model-based details, and analyses were collected. The methodological quality was evaluated using the modified Economic Evaluations Bias (ECOBIAS) checklist. For each of the 22 biases listed in this checklist, studies were categorised into one of four groups: low risk, partial risk, high risk due to inadequate reporting, or high risk. To gauge the overall quality of each study, we computed a composite score by assigning + 2, 0, − 1 and − 2 to each risk category, respectively.

**Results:**

In our analysis of 12 studies, majority adopted a healthcare provider or payer perspective and employed Markov Models with the number of health states varying from 6 to 16. Cost-effectiveness results varied across studies. LAAC displayed a probability exceeding 50% of being the cost-effective option in six out of nine evaluations compared to warfarin, six out of eight evaluations when compared to dabigatran, in three out of five evaluations against apixaban, and in two out of three studies compared to rivaroxaban. The methodological quality scores for individual studies ranged from 10 to − 12 out of a possible 24. Most high-risk ratings were due to inadequate reporting, which was prevalent across various biases, including those related to data identification, baseline data, treatment effects, and data incorporation. Cost measurement omission bias and inefficient comparator bias were also common.

**Conclusions:**

While most studies concluded LAAC to be the cost-effective strategy for stroke prevention in AF, shortcomings in methodological quality raise concerns about reliability and validity of results. Future evaluations, free of these shortcomings, can yield stronger policy evidence.

**Supplementary Information:**

The online version contains supplementary material available at 10.1186/s12962-023-00486-0.

## Introduction

As the healthcare landscape continues to evolve, economic evaluations provide a unique opportunity to furnish essential information to guide policy development, ultimately striving for equitable and effective healthcare delivery. This becomes increasingly pertinent in light of the escalating health expenditure observed worldwide [1]. One example of this relevance is the growing need for stroke prevention among individuals living with atrial fibrillation (AF), the most prevalent sustained cardiac arrhythmia [2, 3].

The conventional approach to stroke prevention in AF involves the administration of oral anticoagulants [4, 5]. An alternative to this lifelong oral drug therapy is the left atrial appendage closure procedure (LAAC), a one-time procedure that entails the percutaneous insertion of a small device into the left atrial appendage of the heart. Despite Clinical Practice Guidelines assigning a Class IIb recommendation to LAAC (usefulness/efficacy is less well established by evidence/ opinion) [4, 5], its use has notably increased in recent years [6, 7].

The surge in the adoption of LAAC has catalysed a proliferation of economic evaluations seeking to estimate its cost-effectiveness, aiming to generate robust evidence to inform and guide pertinent policy decisions. However, the methodological quality of economic evaluations, akin to any other study, plays a pivotal role in shaping its capacity to inform policy decisions. The existing literature has highlighted a proliferation of economic evaluations without necessarily contributing to tangible policy decisions due to various reasons including shortcomings in methodological rigor [8–10].

In a prior review that assessed published economic evaluations estimating the cost-effectiveness of LAAC [11], two out of seven studies were graded with very serious limitations, three with potentially serious limitations, and the remaining two with minor limitations. Since this review, a multitude of economic evaluations comparing LAAC with various oral anticoagulants have been published, yet there remains a gap in establishing the robustness of their methodologies.

This systematic review aims to address this research gap by systematically identifying, evaluating, and consolidating the existing evidence comparing LAAC with oral drugs for stroke prevention in patients with AF. We believe that our paper will offer valuable insights for future health economic evaluations to mitigate common biases frequently encountered in health economic evaluations.

## Materials and methods

We followed Preferred Reporting Items for Systematic reviews and Meta-Analyses (PRISMA) statement in reporting this review (Additional file 1: Table S1) [12]. The review protocol was registered at PROSPERO (registration number CRD42021278841).

### Data sources, search strategy and study selection for the review

We performed a literature search using the electronic databases; PubMed, EMBASE, CINAHL, the Cochrane Central Register of Controlled Trials, and EconLit. Please refer to Additional file 1: Table S3 for our full search strategy.

Two independent reviewers (SH and PS) screened the titles and abstracts of the identified studies using the Rayyan software [13]. Any disagreements were resolved through consultation with a senior author (SK). Full papers presenting original studies conducting model-based economic evaluations to assess the cost-effectiveness of LAAC for stroke prophylaxis in non-valvular AF compared to oral drugs were included. We excluded trial-based evaluations to maintain consistency in our analysis because the checklist we used for quality assessment specifically addresses bias in model-based economic evaluations. The search strategy was limited to studies published in English after the year 2000. Studies that included patients under 18 in their base case population were excluded due to differing management strategies.

### Data extraction

SH and PS independently extracted information on year of publication, study setting, type of economic evaluation, type of economic model used, number and nature of health states, model perspective, the mean age, CHA_2_DS_2_VASc score and HAS-BLED score for the base case population, time horizon, cycle length, annual discount rates, measure of effect, currency type and year for cost, incremental cost-effectiveness ratio (ICER) values, willingness-to-pay threshold (WTP), results for the deterministic and probabilistic sensitivity analyses, main conclusions, main limitations, funding sources and declaration of conflict of interest. These details are presented in Tables 1, 2 for each individual study.

**Table 1:** Characteristics of included studies and their economic evaluations

	Labori et al.	Kawakami et al.	Reddy et al.	Reddy et al.	Ontario HTA series	Lee et al.	Reddy et al.	Freeman et al.	Saw et al.	Micieli et al.	Reddy et al.	Singh et al.
Year of publication	2022	2020	2019	2018	2017	2016	2016	2016	2016	2016	2015	2013
Study setting	Sweden	USA	USA	USA	Canada	USA	Germany	USA	Canada	Canada	USA	Canada
Type of economic evaluation	Cost-utility analysis	Cost-utility analysis	Cost-utility analysis	Cost-utility analysis	Cost-utility analysis	Cost-utility analysis	Cost-utility analysis	Cost-utility analysis	Cost-utility analysis	Cost-utility analysis	Cost-utility analysis	Cost-utility analysis
Modeling technique used	combined decision tree and Morkov model	Markov model	Markov model	Markov model	Markov model	Markov model	Markov model	Markov model	Markov microsimulation model	Markov microsimulation model	Markov model	Markov microsimulation model
Perspective	Swedish healthcare and public sector	US Health care provider	Health insurer	Health insurer	Ontario Ministry of Health and Long Term Care	Healthcare provider	German healthcare system	Health insurer	Healthcare provider	Healthcare provider	Health insurer	Healthcare provider
Compared interventions	LAAC, standard care for AF patients with contraindications to OAC	LAAC following radio-ablation, novel OAC	LAAC, warfarin, novel OAC	LAAC, warfarin, dabigatran, rivaroxaban and apixaban	LAAC, warfarin, apixaban, dabigatran, rivaroxaban	LAAC, Aspirin, (Aspirin + clopidogrel), Warfarin, Dabigatran 110 mg, Dabigatran 150 mg, Apixaban, Rivaroxaban	LAAC, aspirin, apixaban	LAAC, warfarin, dabigatran	LAAC, aspirin	LAAC, warfarin, apixaban, dabigatran, rivaroxaban	LAAC, warfarin, novel OAC	LAAC, warfarin, dabigatran
Base case population	74-year-old patients with nonvalvular AF with contraindications to OAC	65-year-old symptomatic AF patient planned for catheter ablation without contraindication for OAC	70-year-old nonvalvular AF patient without contraindications to OAC	70-year-old nonvalvular AF patient with a history of stroke	nonvalvular AF patients without contraindications for OAC	65-year-old nonvalvular AF patient without any contraindication for anti-thrombotic therapy	70-year-old AF patient with contraindications to warfarin	70 year old Nonvalvular AF patient with no contraindication to OAC	Nonvalvular AF patients at high risk of stroke and with contraindication to OAC	Patients with new onset AF presenting to emergency departments (mean age 68.9) without contraindication to OAC	70-year-old nonvalvular AF without contraindication to OAC	Nonvalvular AF patients without contraindication for OAC
Considered CHA_2_DS_2_VASc score ^a^	4	3	4	7	> 2	Not specified	3	> 1	> 2	Not specified	3.2	> 2
Considered/ mean HAS-BLED score^b^	Not specified	3	1.98	3	0.8	Not specified	3	Not specified	Not specified	Not specified	2	
Measure of effect	QALY	QALY	QALY	QALY	QALY	QALY	QALY	QALY	QALY	QALY	QALY	QALY
Currency type and year	Euro 2020	USD 2020	USD 2017	USD 2016	CAD 2016	USD, year not specified	Euro 2014	USD 2014	CAD 2015	CAD 2012	USD 2015	CAD 2012
Number of health states in the model	11	11	16	16	10	11	16	12	6	10	14	10
Time horizon	Lifetime	10 years	Lifetime (20 years)	Lifetime (20 years)	Lifetime	20 years	20 years	Lifetime	Lifetime	Lifetime	Lifetime (20 years)	Lifetime
Cycle length	1 year	1 year	3 months	3 months	1 month	1 year	3 months	Not mentioned	1 month	1 month	3 months	1 month
Annual discount rate for costs and outcomes	3%	3%	3%	3%	3%	3%	3.50%	3%	5%	5%	3%	5%
Main limitation	How well secondary data matches the patient population in the model	Limitations of primary data	Limitations of primary data	Limitations of primary data	Unavailability of direct clinical evidence comparing LAAC with novel OAC	Limitations of primary data	Limitations of primary data	Limitations of primary data	Lack of standard accepted antithrombotic therapy post- LAAC	Limitations of primary data	Model allowed for only 1 clinical event per 3-month cycle	Limitations of primary data
Funding source	None	None	LAAC manufacturing company	LAAC manufacturing company	Not specified	Not specified	LAAC manufacturing company	Government agencies	None	University sector	Not specified	Federal agency
Conflicts of interest (COI) related to sponsorships by LAAC manufacturing companies	Having no COI declared	Having COI declared	Having COI declared	Having COI declared	Not specified	Having COI declared	Having COI declared	Having COI declared	Having COI declared	Having no COI declared	Having COI declared	Having no COI declared

AF: atrial fibrillation; CAD: Canadian dollars; COI: conflict of interest; DOAC: direct oral anticoagulants; ICER- incremental cost-effectiveness ratio; ICH-intracranial hemorrhage; LAAC-left atrial appendage closure; LAAO- left atrial appendage occlusion; QALY- quality adjusted life years; NOAC: novel oral anticoagulants; NVAF: nonvalvular atrial fibrillation; OAC: oral anticoagulants; PSA- probabilistic sensitivity analysis; UK: United Kingdom; USA: United States of America; USD- United States dollars

^a^Risk of stroke of base case population

^b^Risk of bleeding for the base case population

**Table 2:** Summary of results and conclusions of included model-based economic evaluations presented in primary research papers included in this review

Author and year	Mean effect LAAC	Mean cost of LAAC	Mean effect OAC	Mean cost OAC	ICER per QALY^f^	WTP threshold	Reported probability of the intervention being cost-effective ^e^	Conclusion stated by authors
Warfarin (n = 9)								
Reddy et al. 2019	7.77	USD 44 894	7.17	USD 61 623	USD 48 674^c^ USD 35 051^d^	USD 50 000	LAAC—98%^a^	LAAC is cost-effective and cost saving relative to NOAC and warfarin
Reddy et al. 2018	6.09	USD 55 749	5.66	USD 85 577	Dominant (value not presented)	USD 50 000	LAAC–100%^a^	LAAC is the most cost-effective treatment strategy for secondary prevention of stroke in atrial fibrillation
Ontario HTA	5.66	CAD 40 707	5.60	CAD 24 374	CAD 272 216	CAD 100 000	LAAC—4% Warfarin–0%	LAAC device has higher costs and lower QALYs compared with apixaban, dabigatran and rivaroxaban in patients with nonvalvular AF and no contraindication to OAC
Freeman et al. 2016 (Protect-AF data)	9.94	USD 132 844	7.96	USD 92 190	USD 20 486	USD 50 000	LAAC ~ 90%^b^	The cost effectiveness of LAA closure using PROTECT AF data was in a range generally considered to be cost effective. Using data from PREVAIL, however, LAA closure was dominated by warfarin and dabigatran
Freeman et al. 2016 (Prevail data)	8.44	USD 120 977	8.54	USD 73 077	Dominated (value not presented)	USD 50 000	Warfarin ~ 78%^b^ LAAC–9%	
Lee et al. 2016	10.99	USD 37 789	9.45	USD 28,090	USD 6 298	USD 50 000	LAAC–86%^a^	LAAO was cost-effective compared to all tested OAC
Micieli et al. 2016	5.21	CAD 21 789	5.13	CAD 15 776	Considered warfarin as the reference	CAD 50 000	LAAC ~ 30%^b^ Warfarin ~ 12%	Apixaban is the preferred long-term strategy
Reddy et al. 2015	8.03	USD 31 198	7.39	USD 49 946	USD 42 994	USD 50 000	LAAC–98%^a^	Both NOAC and LAAC with the Watchman device were cost-effective relative to warfarin, but LAAC was also found to be cost-effective and to offer better value relative to NOAC
Singh et al. 2013	4.68	CAD 27 003	4.55	CAD 21 429	CAD 41 565	CAD 50 000	Warfarin ~ 44% LAAC ~ 43%^b^	LAAC is cost-effective compared with warfarin therapy
Novel OAC as a class (n = 4)								
Labori et al. 2022	7.11	Healthcare perspective: € 19 032 Public sector perspective: € 21 029	6.12	Healthcare perspective: € 15 022 Public sector perspective: € 31 281	Healthcare perspective: € 4047 Public sector perspective: LAAC is dominant	Euro 45 829	Healthcare perspective: % Not specified Public sector perspective: LAAC—99%	LAAC is cost-effective than OAC from both healthcare and public sector perspective
Kawakami et al. 2020	6.13	USD 29 027	6.03	USD 27 896	USD 11 072	USD 50 000	(LAAC + CA) > 70%^a^	Combined CA and LAAC procedure may be a cost-effective therapeutic option
Reddy et al. 2019	7.77	USD 44 894	7.48	USD 77 023	Dominant (value not presented)	USD 50 000	LAAC–95%^a^	LAAC is cost-effective and cost saving relative to NOAC and warfarin
Reddy et al. 2015	8.03	USD 31 198	7.68	USD 61 701	USD 48 446 relative to warfarin	USD 50 000	LAAC–95%^a^	Both NOAC and LAAC with the Watchman device were cost-effective relative to warfarin, but LAAC was also found to be cost-effective and to offer better value relative to NOAC
Dabigatran (n = 8)								
Reddy et al. 2018	6.09	USD 55 749	5.84	USD 87636	Dominant (value not presented)	USD 50 000	LAAC–90%^a^	LAAC is the most cost-effective treatment strategy for secondary prevention of stroke in atrial fibrillation
Ontario HTA	5.66	CAD 40 707	5.81	CAD 25 694	Dominated (value not presented)	CAD 100 000	Dabigatran–47% LAAC–4%	LAAC device has higher costs and lower QALYs compared with apixaban, dabigatran and rivaroxaban in patients with nonvalvular AF and no contraindication to OAC
Lee et al. 2016 (Dabigatran 110 mg)	10.99	USD 37 789	8.76	USD 42 712	Dominated (value not presented)	USD 50 000	LAAC—86%^a^	LAAO was cost-effective compared to all tested OAC
Lee et al. 2016 (Dabigatran 150 mg)	10.99	USD 37 789	9.00	USD 43 946	Dominated (value not presented)	USD 50 000	LAAC–86%^a^	LAAO was cost-effective compared to all tested OAC
Freeman et al. 2016 (Protect-AF data)	9.94	USD 132 844	8.28	USD 94 072	USD 23 422	USD 50 000	LAAC ~ 90%^b^	The cost effectiveness of LAA closure using PROTECT AF data was in a range generally considered to be cost effective. Using data from PREVAIL, however, LAA closure was dominated by warfarin and dabigatran
Freeman et al. 2016 (Prevail data)	8.44	USD 120 977	8.59	USD 83 746	Dominated (value not presented)	USD 50 000	Dabigatran ~ 11%^b^ LAAC–9%	
Micieli et al. 2016	5.21	CAD 21 789	5.18	CAD 20 794	Dominated (value not presented)	CAD 50 000	LAAC ~ 30%^b^ Dabigatran–0%	Apixaban is the preferred long-term strategy
Singh et al. 2013	4.68	CAD 27 003	4.64	CAD 25 760	CAD 46 560 compared to warfarin	CAD 50 000	LAAC ~ 43%^b^ Dabigatran ~ 10%	LAAC is cost-effective compared with warfarin therapy
Apixaban (n = 5)								
Reddy et al. 2018	6.09	USD 55749	5.82	USD 85426	Dominant (value not presented)	USD 50 000	LAAC–95%^a^	LAAC is the most cost-effective treatment strategy for secondary prevention of stroke in atrial fibrillation
Ontario HTA	5.66	CAD 40 707	5.82	Dominated (value not presented)	USD -80 758	CAD100 000	Apixaban–48% LAAC–4%	LAAC device has higher costs and lower QALYs compared with apixaban, dabigatran and rivaroxaban in patients with nonvalvular AF and no contraindication to OAC
Micieli et al. 2016	5.21	CAD 21 789	5.25	CAD 19 156	CAD 28 167 compared to warfarin	CAD 50 000	Apixaban–40%^a^ LAAC ~ 30%	Apixaban is the preferred long-term strategy
Reddy et al. 2016	4.82	€ 15 837	4.59	€ 18 869	€ 9040	€ 30 000	LAAC–94%^a^	LAAC with the Watchman device is a cost-effective and cost-saving solution
Lee et al. 2016	10.99	USD 37 789	9.40	USD 53 315	Dominated (less costly, more effective)	USD 50 000	LAAC–86%^a^	LAAO was cost-effective compared to all tested OAC
Rivaroxaban (n = 3)								
Ontario HTA	5.66	CAD 40 707	5.74	CAD 30 530	Dominated (value not presented)	CAD 100 000	Rivaroxaban–1% LAAC–4%	LAAC device has higher costs and lower QALYs compared with apixaban, dabigatran and rivaroxaban in patients with nonvalvular AF and no contraindication to OAC
Lee et al. 2016	10.99	USD 37 789	9.86	USD 51 064	Dominated (less costly, more effective)	USD 50 000	LAAC–86%^a^	LAAO was cost-effective compared to all tested OAC
Micieli et al. 2016	5.21	CAD 21 789	5.21	CAD 18 280	CAD 31 300 compared to warfarin	CAD 50 000	Rivaroxaban ~ 12% LAAC ~ 30%^b^	Apixaban is the preferred long-term strategy

CAD: Canadian dollar, CA: catheter ablation; ICER: incremental cost-effectiveness ratio; LAAC: left atrial appendage closure; NOAC: novel oral anticoagulants; OAC: oral anticoagulant; PSA: probabilistic sensitivity analysis; WTP: willingness to pay; US$: US dollar; €: Euro

^a^PSA probability reported in the paper

^b^PSA probability extracted from the cost-effectiveness acceptability curve presented in the paper

^c^value presented in the abstract of the paper

^d^value presented in the main text of the paper

^e^When the probability of LAAC being cost-effective at the given willingness-to-pay threshold is less than 50%, the probability of the compared OAC being cost-effective is presented

^f^LAAC considered as the intervention and compared with an oral drug unless specified otherwise

### Methodological quality assessment

We employed the Risk of Bias in model-based economic evaluation (ECOBIAS) checklist to guide our quality assessment [14] to assess the methodological quality of the included economic evaluations. Developed in accordance with best practice guidelines in the field of health economics, this checklist comprises 22 items that evaluate both general bias in health economic evaluations and model-specific bias. This approach distinguishes ECOBIAS from other checklists, which may focus on reporting quality [15] or broader good practice guidelines that are not primarily focused on bias [16, 17].

We used the ‘questions to consider’ provided within the ECOBIAS checklist to assign one of four ratings for each bias: low risk, partial risk, high risk due to inadequate reporting, or high risk (Please refer to Additional file 1: Table S4). If we could not find adequate information in the main text or supplementary files of a study to satisfactorily address these questions, a rating of high-risk due to inadequate reporting was assigned. When we had sufficient information available, we assessed the risk of bias based on the responses to the ‘questions to consider’. A high-risk rating was assigned when more than 50% of the ‘questions to consider’ received negative answers (e.g., no, not justified). Conversely, if this percentage was less than 50%, we assigned a partial risk rating for the relevant bias. A low-risk rating was given when we could answer all the ‘questions to consider’ positively (e.g., yes, justified) based on the available information.

Additionally, we computed composite scores at both the study and item levels by allocating scores as follows: + 2 for low risk, 0 for partial risk, − 1 for high risk due to inadequate reporting, and − 2 for high risk.

Two authors, SH and RN, conducted individual assessments for each study, and any discrepancies were resolved through discussions between the two authors. A visual representation of the ratings assigned to each study can be found in Table 3, while a comprehensive description of the risk of bias assessment is provided in Additional file 1: Table S4.

## Results

### Study selection

**Fig. 1:**
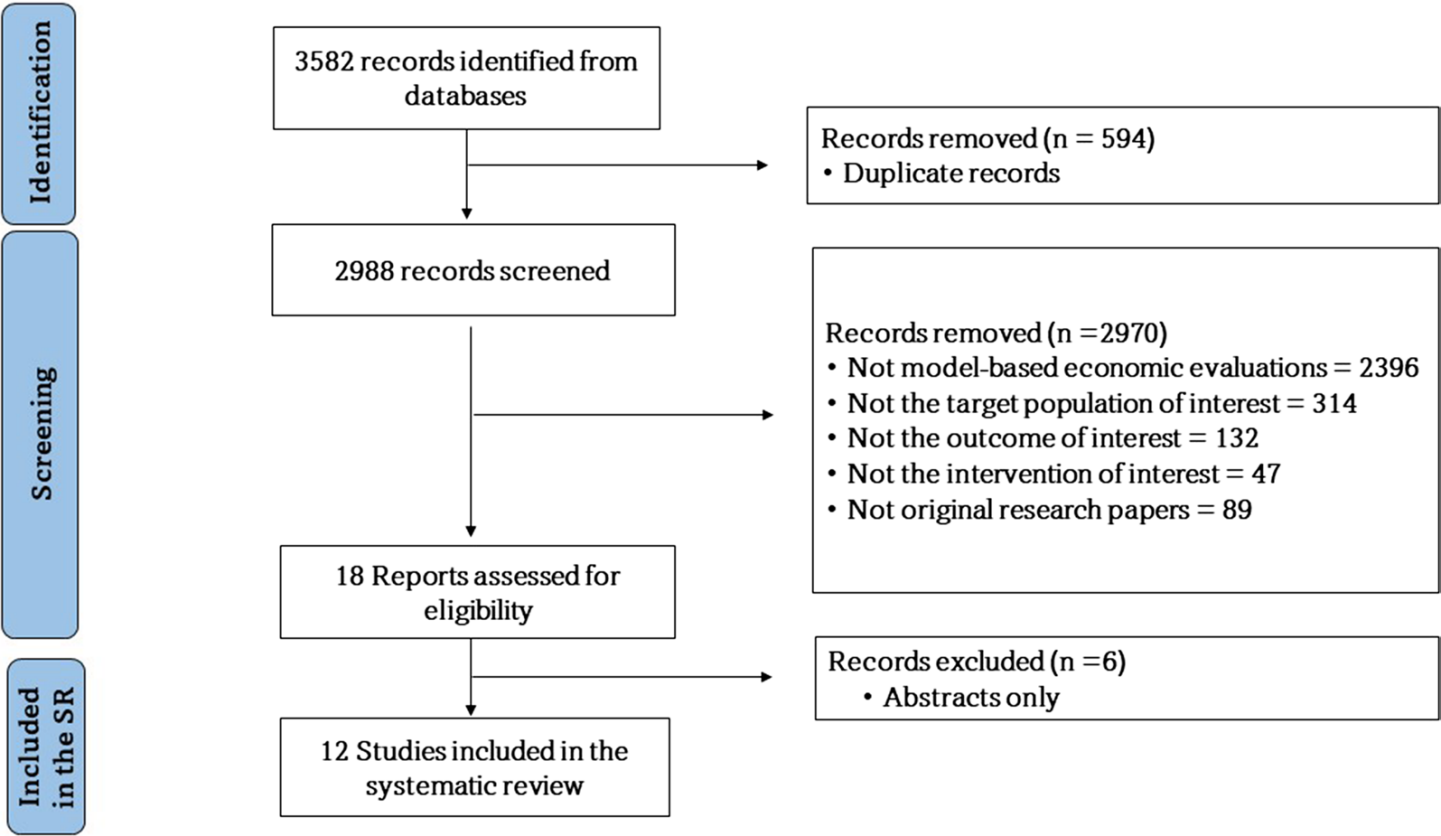
PRISMA flow chart on study selection for the review [26]

Out of the 3580 studies identified through the search strategy, 12 were included in the review [11, 18–28] (Fig. 1).

### Main study and economic model characteristics

All the studies included in our analysis focused on elderly patients of both genders who had non-valvular AF in high-income country settings. The age range of the base-case populations typically fell between 65 and 70 years. According to AF guidelines, patients with AF who are at a higher risk of stroke, as determined by the CHADS_2_VASc score, are eligible for stroke prophylaxis. Recommendations specify a score greater than 1 for males and greater than 2 for females [4]. Two studies we reviewed did not specify the CHADS_2_VASc score for their base-case populations [21, 24]. Freeman et al. [23] considered a score greater than 1, regardless of gender, in the base case population. The remaining studies adopted a score greater than 2. Notably, Reddy et al. [20] studied a population of AF patients with a history of stroke episodes, significantly increasing their risk of subsequent strokes. The mean CHADS_2_VASc score of this study was 7.

All the studies employed Markov state transition models for their analyses, adopting a healthcare provider or payer perspective. The number of health states within these models varied, ranging from 6 to 16 states. These states included critical outcomes in AF, such as myocardial infarction, minor and major stroke, and minor and major bleeding. Notably, two studies excluded myocardial infarction from their analyses stating a lack of available clinical input data [18, 28].

Regarding time horizons, one study [18] adopted a 10-year horizon and other studies utilised a lifetime horizon. Cycle lengths also exhibited variation, with studies employing a 1 month cycle (n = 4), a 3 month cycle (n = 4), and a 1 year cycle (n = 3). One study [23] did not specify the cycle length used in their analysis.

All the studies included in the review provided data on the mean cost, mean effect, and Incremental Cost-Effectiveness Ratio (ICER) values for the LAAC and the comparators, alongside the specified willingness-to-pay (WTP) threshold. Most studies also conducted both deterministic and probabilistic sensitivity analyses to assess parameter uncertainty. A common limitation discussed in these papers was the unavailability of trial data for model input parameters. Table 1 summarises the key characteristics of the included studies.

### Results of cost-effectiveness analyses

Warfarin was the most frequently compared oral drug against LAAC (n = 9). In six of these evaluations (67%), LAAC had a more than 50% probability of being cost-effective compared to warfarin [19–21, 23, 26, 27]. In the majority of evaluations with dabigatran (6 out of 8 studies) [20, 21, 23, 24, 27], apixaban (3 out of 5 studies) [11, 20–22, 24], and rivaroxaban (2 out of 3 studies) [11, 21, 24], LAAC emerged as the cost-effective intervention in 50% or more of the times. [20, 21, 23, 24, 27]. Table 2 provides a summary of the results from the cost-effectiveness analyses for each of the compared oral anticoagulants.

### Results of the methodological quality assessment

The composite score for individual studies based on the risk of bias assessment, varied from 10 to − 12, with a maximum possible score of 24. All studies received a low-risk rating for several biases, which included double counting bias, ordinal ICER bias, no treatment-comparator bias, wrong model bias, and bias related to quality-of-life weights (utilities).

Partial and high-risk ratings were commonly assigned to the remaining biases in the studies. All studies were graded as having a high risk for cost measurement omission bias because they did not consider implementation costs for LAAC or oral anticoagulants. Additionally, three studies [18, 23, 27] received a high-risk rating for inefficient comparator bias as their models did not account for all therapeutic modalities within standard care.

In most studies, high-risk grades were assigned for various other biases mainly due to inadequate reporting. Notably, a significant number of studies (11 out of 12) either omitted or provided limited information regarding their approach to identifying data sources for the model and the justification for their chosen approach. This made them vulnerable to receiving high-risk ratings for bias related to data identification (item 16 in the ECOBIAS checklist). Only the study by Labori and colleagues [28] provided a detailed account of their approach to data source identification and justifications.

Likewise, all studies were assigned high-risk ratings for bias related to baseline data (item 17 in the ECOBIAS checklist) because they did not provide specific details about the conversion of rates into transition probabilities. Concerning bias related to treatment effects, some studies did not offer any information about the extrapolation methods employed beyond the trial period [15, 18–20]. For those that did include extrapolations, they often did not provide justifications or explore alternative assumptions for extrapolation through sensitivity analyses, as recommended by guidelines [16, 29].

Furthermore, all studies were rated as having a high risk due to inadequate reporting for bias related to data incorporation (item 20 in the ECOBIAS checklist). While each study referenced the sources of model input data, it was not clear how the values used in the model were derived from these referenced sources. For instance, an Australian study [18] referred to two cost-effectiveness studies conducted in France [30] and the United States [31] for costs associated with stroke and intracranial haemorrhage, but the study did not explain how the values used in their own analysis were derived from these referenced sources. This was a prevalent issue observed consistently across all the studies.

I Insufficient reporting in these studies rendered them vulnerable to various other biases, including narrow perspective bias, intermittent data collection bias, double counting bias, inappropriate discounting bias, limited sensitivity analysis bias, reporting and dissemination bias, limited scope bias, and bias related to internal consistency.

A comprehensive description of the risk of bias assessment is provided in Additional file 1: Table S4. Additionally, Table 3 visually presents the bias risk for each study.

**Table 3:**
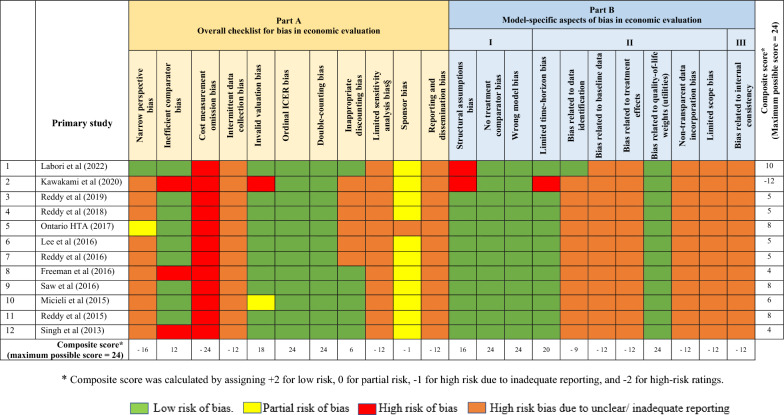
Risk of Bias assessment using ECOBIAS checklist.

## Discussion

The 12 studies included in this review aimed to assess the cost-effectiveness of LAAC as a stroke prevention strategy for individuals living with AF, in comparison to oral drugs. A large majority (11 out of 12) of the studies adopted a healthcare provider or payer perspective. All of these studies employed Markov models, with the number of health states in the models ranging from 6 to 16. The findings varied across studies regarding the cost-effectiveness of LAAC compared to the comparator. Most evaluations estimated that LAAC would be the cost-effective stroke prevention strategy in 50% or more of the times against the compared oral anticoagulant.

Our finding of varying levels of cost-effectiveness for LAAC is consistent with current knowledge that the cost-effectiveness of the LAAC device compared to novel oral anticoagulants remains uncertain [11]. Published literature also suggest that some economic models may have overestimated the benefits of LAAC compared to novel oral anticoagulants [32].

The methodological quality of the 12 studies included in our analysis exhibited variability, with the composite score derived from their bias ratings ranging from 10 to − 12. To the best of our knowledge, the only existing evidence regarding the methodological quality of economic evaluations assessing LAAC compared to oral anticoagulants comes from a health technology assessment (HTA) conducted by Ontario Health [11]. This HTA covered seven of the 12 studies included in our review and yielded conclusions that closely align with our findings. Within these overlapping studies, the Ontario review categorised two [21, 23] out of the seven as having very serious limitations, three [24, 26, 27] as having potentially serious limitations, and two as having minor limitations [22, 25] based on an eleven-item checklist developed by the authors in accordance with best practice guidelines. Our risk of bias assessments was compatible with their ratings for most items, with the exception in the 'inclusion of all important and relevant costs' category for two studies [22, 26]. While the Ontario review reported that these two studies incorporated all important and relevant costs, our grading indicated a 'high risk due to unclear reporting' for cost measurement omission bias, as these two studies did not include any implementation costs for the interventions or follow-up care costs for LAAC.

In our efforts to enhance the quality of model-based economic evaluations in the future, we aim to address some of the common biases and concerns that we have identified through our review.

### Inadequate reporting

Our review underscores that a significant portion of the high-risk bias arises from unclear or insufficient reporting, as demonstrated in Table 3. Frequently, overlooked or insufficiently reported elements encompass a wide range of aspects including the techniques employed to identify data sources, synthesising baseline and treatment effectiveness data, data incorporation, methods for validating the model or methods for handling methodological, structural uncertainty and heterogeneity. Our finding of studies having a high risk for several biases due to inadequate reporting is in line with prior reviews of cost-effectiveness analyses [33, 34].

The problem of insufficient reporting in economic evaluations has been recognised for quite some time [33, 35, 36] leading to the formulation of various guidelines and checklists aimed at improving reporting standards [15, 16]. However, findings from our review highlights that the issue remains unresolved. Utilising the ‘questions to consider’ outlined in the ECOBIAS checklist for each bias could serve as a valuable guide for authors to evaluate the comprehensiveness of their reporting.

### Appropriateness of data sources

Most studies in this review utilised PROTECT-AF and PREVAIL trials to derive treatment effectiveness data, given that these were the sole trials comparing LAAC to any oral drugs at the time. Ensuring a robust representation of underlying effectiveness data is a crucial consideration in economic evaluations [37].

While acknowledging the prevalent use of trial data in health economic modelling, it is important to note that they do not always offer a comprehensive representation of the current evidence base. Trials are acknowledged to lack generalisability due to strict patient criteria, dosing protocols, follow-up intensity, and supportive care use [38–40]. In 2007, ISPOR endorsed the use of real-world evidence, defined as economic, clinical, or patient-centred information from pragmatic trials, registries, administrative data, health surveys, and electronic or paper records, over randomised controlled trials for coverage and reimbursement decisions [41].

Observational data from registries and follow-up studies for LAAC [42, 43] and novel oral anticoagulants [44, 45] possess the potential to exert a transformative influence on the determination of treatment efficacy, thereby potentially reshaping the cost-effectiveness assessment of LAAC in the domain of stroke prevention. For example, the meta-analysis [46] used by Labori and colleagues [28] to retrieve treatment effects for LAAC examined 29 studies, including trials, observational studies and registries. It is noteworthy that other studies which utilised only trial data did not present a rationale for the omission of observational studies from the process of data synthesis despite best practice guidelines suggesting otherwise [47]. Furthermore, apprehensions regarding the methodological rigor of the PROTECT-AF and PREVAIL trials [48–50] diminish their suitability as the sole source of data for effectiveness measures. We are of the opinion that combining both trial and observational studies related to the research question would have provided the best available evidence base from which to draw parameter estimates.

We also observed that, in certain studies, the suitability of the data sources used to obtain model input parameters appeared to be questionable. For instance, Kawakami et al. [18] examined a base case population of individuals undergoing LAAC along with catheter ablation. However, participants in the PROTECT-AF and PREVAIL trials which served as the primary data sources for treatment effect did not undergo catheter ablation.

Similarly, in the economic model by Reddy et al. [20] the cost-effectiveness of LAAC was examined within a cohort of AF patients who had experienced a prior stroke, exhibiting a mean CHA_2_DS_2_ score of 7. Having a CHA_2_DS_2_ score of 7 indicate a very high likelihood for a subsequent stroke. It is worth noting that the corresponding scores in the PROTECT-AF and PREVAIL trials, which provided the exclusive treatment effect data sources, were only 2.2 ± 1.2 and 2.3 ± 1.2 respectively [51].

Furthermore, three studies [22, 25, 28] centred their base case populations on individuals with contraindications for oral anticoagulants within their economic models. On the contrary, the PROTECT-AF and PREVAIL trials which were used for effectiveness data excluded individuals with contraindications for oral anticoagulants [52].

The outcomes of an economic evaluation may stray from effectively addressing the specific research question at hand when the model inputs do not closely align with the pertinent context. This aspect has been widely emphasised in the literature [36, 37, 52, 53]. We believe that allocating higher priority to the suitability of data sources is essential for enhancing the reliability and validity of results derived from economic evaluations. We acknowledge that such appropriate data may not be always available. Nevertheless, we emphasise the significance of a deliberate decision-making process for policymakers and modelers when choosing between utilising available data, even if it does not align well with the research context, and waiting for more appropriate data. It is important to recognise that more fitting data can be derived from real-world evidence, such as registry data and observational studies. Superior evidence from such models enhances the utility of economic evaluations in guiding policy decisions and represents a more efficient allocation of limited research resources.

### Inefficient comparator bias and cost measurement omission bias

The emphasise on the most appropriate point of reference being the ‘current standard of care’ or the therapeutic modalities that hold the widest usage within the pertinent jurisdiction is strong among established guidelines governing economic evaluations [37, 53]. While warfarin, dabigatran, apixaban, and rivaroxaban are frequently prescribed for stroke prevention in AF patients without contraindications, only six out of nine studies [11, 19–21, 24, 26] concentrating on this patient subgroup undertook comprehensive comparisons of all prevalent treatments alongside LAAC. Neglecting to encompass all pertinent options within the analysis for a specific patient cohort is likely to result in a partial evaluation [53], potentially impeding its utility within the decision-making process. A review of pharmaceutical reimbursement submissions in Australia [54] found that 6% of the studies exhibited uncertainty in selecting or using inappropriate comparators [54].

Another prevalent pitfall in economic evaluations is the omission of implementation costs [55, 56]. None of the evaluations included in our review accounted for implementation costs associated with compared interventions. These costs entail expenses for acquiring capital equipment, training medical and other staff, supplying medical consumables and reagents, and initiating and maintaining quality control measures. This oversight can result in an underestimation of costs, potentially leading to overly optimistic cost-effectiveness estimates [57]. Existing frameworks [57] offer a valuable means to assess essential implementation costs, and pertinent data can be collected through related cost-of-illness studies [58] and qualitative interviews with stakeholders [59].

### Strengths and limitations

Our review has a significant strength in that we utilised a checklist that appraises the bias related to economic evaluation. This checklist was adapted from the best practice guidelines in the field of health economics, and it provided a framework for critically reviewing the economic evaluations considered in this article. However, we acknowledge that there was room for subjective interpretation, which may have influenced our assessment of the methodological quality. To minimise this potential bias, we took a rigorous approach by having two independent reviewers appraise the quality of primary studies, and any disagreements were resolved through discussion and consensus.

We have also come to recognise that certain questions outlined in the ECOBIAS checklist which was used to guide the quality assessment might not always be applicable to model-based economic evaluations. For instance, Item 11 addresses reporting and dissemination bias by inquiring, "Has the study been listed in a trial register? Have all results been reported according to the study protocol?". Similarly, Item 10 pertains to sponsor bias and queries the free accessibility of the study protocol. Although these inquiries are of less relevance in model-based economic evaluations, we adhered to the prescribed methods for quality assessment, potentially resulting in the assignment of high-risk grades for these biases than may be warranted.

Conversely, we observed a lack of questions aimed at assessing the appropriateness of data sources for the decision context and research question within the checklist. Given that this is a pivotal factor influencing methodological quality, we recommend the incorporation of inquiries concerning the appropriateness of data sources for the research context into the ECOBIAS checklist [37].

## Conclusions and recommendations

While most studies concluded LAAC to be the cost-effective option for stroke prevention in AF, shortcomings in methodological quality raise concerns about the validity of results.

Inadequate reporting led to the classification of numerous studies as having a high risk for multiple biases. Their effects could potentially inflate or deflate the cost-effectiveness of LAAC, contingent upon how they influenced the cost and effects of the interventions compared within the model. We suggest that the questions presented in the ECOBIAS checklist can serve as a valuable tool for authors to gauge the sufficiency of their reporting, as it continues to be a prevalent concern within health economic evaluations. Additionally, not utilising most appropriate data for the research context may have yielded less reliable results.

Cost omission bias which was observed in all studies is likely to have skewed the cost-effectiveness results in favour of LAAC, as the omitted costs were predominantly associated with this procedure. Integrating implementation costs for the interventions being evaluated will likely generate results that better reflect the complexities of real-world settings.

Furthermore, we recommend using real-world evidence such as registry data, observational data and survey data in model input parameters is likely to improve the validity and reliability of results. Future evaluations should consider all commonly used stroke prevention strategies within usual care as comparators to provide a more comprehensive assessment for evidence-informed decision-making.

Addressing these methodological pitfalls in future evaluations can generate robust evidence to inform policy decisions.

### Supplementary Information

**Additional file 1.** PRISMA checklist, Search strategy, Quality assessment using ECOBIAS checklist, References.

## Data Availability

All data leading to the production of results are provided in the paper and supplementary materials.

## Abbreviations

AF: Atrial fibrillation
ECOBIAS: Modified Economic Evaluations Bias checklist
HTA: Health Technology assessment
ICER: Incremental cost-effectiveness ratio
LAAC: Left atrial appendage occlusion
PROTECT-AF: WATCHMAN left atrial appendage system for embolic protection in patients with atrial fibrillation trial
REVEAL: Watchman Left Atrial Appendage Closure device in patients with atrial fibrillation versus long-term warfarin therapy trial
WTP: Willingness to pay
