# Supplementary material for: Cost-effectiveness of left atrial appendage closure for stroke prevention in atrial fibrillation: a systematic review appraising the methodological quality
Source: Cost Eff Resour Alloc. 2023 Oct 23;21:76. doi: 10.1186/s12962-023-00486-0 (PMC10591401; doi:10.1186/s12962-023-00486-0)
Supplement: Supplementary file 1 — Additional file 1. PRISMA checklist, Search strategy, Quality assessment using ECOBIAS checklist, References. [file 12962_2023_486_MOESM1_ESM.pdf]

Additional file

Cost-effectiveness of left atrial appendage closure for stroke prevention in atrial fibrillation: a systematic review appraising the methodological quality.

Sumudu A. Hewage[1,2], Rini Noviyani[3], David Brain[1], Pakhi Sharma[1], William Parsonage[1,4], Steven M. McPhail[1,5], Adrian Barnett[1], Sanjeewa Kularatna[1]

1. Australian Centre for Health Services Innovation and Centre for Healthcare Transformation, Queensland University of Technology, Australia.
2. Directorate of Public Health, Ministry of Health, Colombo, Sri Lanka.
3. Department of Pharmacy, Udayana University, Bali, Indonesia.
4. Cardiology department, Royal Brisbane and Women's Hospital, Queensland, Australia
5. Digital Health and Informatics Directorate, Metro South Health, Queensland, Australia.

Table s1. Prisma for Abstracts Checklist

Section and Topic	Item #	Checklist item	Reported (Yes/No)
**TITLE**			
Title	1	Identify the report as a systematic review.	Yes
**BACKGROUND**			
Objectives	2	Provide an explicit statement of the main objective(s) or question(s) the review addresses.	Yes
**METHODS**			
Eligibility criteria	3	Specify the inclusion and exclusion criteria for the review.	Yes
Information sources	4	Specify the information sources (e.g. databases, registers) used to identify studies and the date when each was last searched.	Yes
Risk of bias	5	Specify the methods used to assess risk of bias in the included studies.	Yes
Synthesis of results	6	Specify the methods used to present and synthesise results.	Yes
**RESULTS**			
Included studies	7	Give the total number of included studies and participants and summarise relevant characteristics of studies.	Yes
Synthesis of results	8	Present results for main outcomes, preferably indicating the number of included studies and participants for each. If meta-analysis was done, report the summary estimate and confidence/credible interval. If comparing groups, indicate the direction of the effect (i.e. which group is favoured).	Yes
**DISCUSSION**			
Limitations of evidence	9	Provide a brief summary of the limitations of the evidence included in the review (e.g. study risk of bias, inconsistency and imprecision).	Yes
Interpretation	10	Provide a general interpretation of the results and important implications.	Yes
**OTHER**			
Funding	11	Specify the primary source of funding for the review.	No, but details are available within the main text.
Registration	12	Provide the register name and registration number.	No, but details are available within the main text.

Table s2. PRISMA Checklist

Section and Topic	Item #	Checklist item	Location where item is reported
**TITLE**			
Title	1	Identify the report as a systematic review.	Title
**ABSTRACT**			
Abstract	2	See the PRISMA 2020 for Abstracts checklist.	Abstract
**INTRODUCTION**			
Rationale	3	Describe the rationale for the review in the context of existing knowledge.	4th paragraph of Introduction
Objectives	4	Provide an explicit statement of the objective(s) or question(s) the review addresses.	5th paragraph of the Introduction.
**METHODS**			
Eligibility criteria	5	Specify the inclusion and exclusion criteria for the review and how studies were grouped for the syntheses.	'Data sources, search strategy and study selection for the review' sub-section
Information sources	6	Specify all databases, registers, websites, organisations, reference lists and other sources searched or consulted to identify studies. Specify the date when each source was last searched or consulted.	'Data sources, search strategy and study selection for the review' sub-section
Search strategy	7	Present the full search strategies for all databases, registers, and websites, including any filters and limits used.	Supplementary file
Selection process	8	Specify the methods used to decide whether a study met the inclusion criteria of the review, including how many reviewers screened each record and each report retrieved, whether they worked independently, and if applicable, details of automation tools used in the process.	'Data sources, search strategy and study selection for the review' sub-section
Data collection process	9	Specify the methods used to collect data from reports, including how many reviewers collected data from each report, whether they worked independently, any processes for obtaining or confirming data from study investigators, and if applicable, details of automation tools used in the process.	'Data extraction' sub-section
Data items	10a	List and define all outcomes for which data were sought. Specify whether all results that were compatible with each outcome domain in each study were sought (e.g. for all measures, time points, analyses), and if not, the methods used to decide which results to collect.	'Data extraction' and 'methodological quality assessment' sub-sections
	10b	List and define all other variables for which data were sought (e.g. participant and intervention characteristics, funding sources). Describe any assumptions made about any missing or unclear information.	'Data extraction' sub-section
Study risk of bias assessment	11	Specify the methods used to assess risk of bias in the included studies, including details of the tool(s) used, how many reviewers assessed each study and whether they worked independently, and if applicable, details of automation tools used in the process.	Sub-section 'methodological quality assessment'
Effect measures	12	Specify for each outcome the effect measure(s) (e.g. risk ratio, mean difference) used in the synthesis or presentation of results.	'Data extraction' sub-section
Synthesis methods	13a	Describe the processes used to decide which studies were eligible for each synthesis (e.g. tabulating the study intervention characteristics and comparing against the planned groups for each synthesis (item #5)).	'Data extraction' sub-section
	13b	Describe any methods required to prepare the data for presentation or synthesis, such as handling of missing summary statistics, or data conversions.	Not applicable

Section and Topic	Item #	Checklist item	Location where item is reported
	13c	Describe any methods used to tabulate or visually display results of individual studies and syntheses.	Sub-sections “data extraction’ and ‘methodological quality assessment’
	13d	Describe any methods used to synthesize results and provide a rationale for the choice(s). If meta-analysis was performed, describe the model(s), method(s) to identify the presence and extent of statistical heterogeneity, and software package(s) used.	‘Sub-section ‘Methodological quality assessment’. Meta-analysis was not performed.
	13e	Describe any methods used to explore possible causes of heterogeneity among study results (e.g. subgroup analysis, meta-regression).	Not applicable
	13f	Describe any sensitivity analyses conducted to assess robustness of the synthesized results.	Not applicable
Reporting bias assessment	14	Describe any methods used to assess risk of bias due to missing results in a synthesis (arising from reporting biases).	Not applicable
Certainty assessment	15	Describe any methods used to assess certainty (or confidence) in the body of evidence for an outcome.	Not applicable
**RESULTS**			
Study selection	16a	Describe the results of the search and selection process, from the number of records identified in the search to the number of studies included in the review, ideally using a flow diagram.	Sub-section ‘Study selection’ and Figure 1
	16b	Cite studies that might appear to meet the inclusion criteria, but which were excluded, and explain why they were excluded.	Sub-section ‘Study selection’ and supplementary file table s
Study characteristics	17	Cite each included study and present its characteristics.	Sub-section ‘Main study and economic model characteristics’
Risk of bias in studies	18	Present assessments of risk of bias for each included study.	Sub-section ‘methodological quality assessment’
Results of individual studies	19	For all outcomes, present, for each study: (a) summary statistics for each group (where appropriate) and (b) an effect estimate and its precision (e.g. confidence/credible interval), ideally using structured tables or plots.	Table 1
Results of syntheses	20a	For each synthesis, briefly summarise the characteristics and risk of bias among contributing studies.	Sub-sections ‘Main study and economic model characteristics’ and ‘methodological quality assessment’
	20b	Present results of all statistical syntheses conducted. If meta-analysis was done, present for each the summary estimate and its precision (e.g. confidence/credible interval) and measures of statistical heterogeneity. If comparing groups, describe the direction of the effect.	Table 2. meta-analysis was not conducted.
	20c	Present results of all investigations of possible causes of heterogeneity among study results.	Not applicable.
	20d	Present results of all sensitivity analyses conducted to assess the robustness of the synthesized results.	Not applicable.
Reporting biases	21	Present assessments of risk of bias due to missing results (arising from reporting biases) for each synthesis assessed.	Not applicable.
Certainty of evidence	22	Present assessments of certainty (or confidence) in the body of evidence for each outcome assessed.	Not applicable.
**DISCUSSION**			
Discussion	23a	Provide a general interpretation of the results in the context of other evidence.	First two paragraphs of the Discussion.
	23b	Discuss any limitations of the evidence included in the review.	Throughout the Discussion
	23c	Discuss any limitations of the review processes used.	Subs-section ‘Strengths and Limitations’

Section and Topic	Item #	Checklist item	Location where item is reported
	23d	Discuss implications of the results for practice, policy, and future research.	Throughout the Discussion section
**OTHER INFORMATION**			
Registration and protocol	24a	Provide registration information for the review, including register name and registration number, or state that the review was not registered.	First paragraph of the 'Materials and methods' sub-section.
	24b	Indicate where the review protocol can be accessed, or state that a protocol was not prepared.	First paragraph of the 'Materials and methods' sub-section.
	24c	Describe and explain any amendments to information provided at registration or in the protocol.	Not presented.
Support	25	Describe sources of financial or non-financial support for the review, and the role of the funders or sponsors in the review.	After the main text under the heading 'Funding'
Competing interests	26	Declare any competing interests of review authors.	After the main text under the heading 'Conflict of interest'
Availability of data, code and other materials	27	Report which of the following are publicly available and where they can be found: template data collection forms; data extracted from included studies; data used for all analyses; analytic code; any other materials used in the review.	A meta-analysis was not performed.

From: Page MJ, McKenzie JE, Bossuyt PM, Boutron I, Hoffmann TC, Mulrow CD, et al. The PRISMA 2020 statement: an updated guideline for reporting systematic reviews. BMJ 2021;372:n71. doi: 10.1136/bmj.n71

For more information, visit: http://www.prisma-statement.org/

Table s3. Search strategy of the review

Theme	#	Search strategy
Atrial fibrillation related terms	1	atrial fibrillation [MeSH] OR af OR a-fib OR afib OR atrial OR atrium OR auricular OR supraventricular OR supra-ventricular OR cardiac OR heart
	2	fibril* OR arrhythm* OR arrythm* OR dysrhythm* OR tachyarrhyth* OR tachy-arrhyth* OR tachyarrythm* OR tachy-arrythm* OR tachycardia OR rhythm abnormal* OR rate abnormal*
	3	#1 AND #2
Stroke related terms	4	Stroke [MeSH] OR strokes * OR Cerebrovascular Accident OR Cerebrovascular Accidents OR Cerebrovascular Apoplexy OR Brain Vascular Accident OR Brain Vascular Accidents OR Acute Cerebrovascular Accident OR Acute Cerebrovascular Accidents
Economic evaluation related terms	5	cost benefit analysis [MeSH] OR cost benefit analysis OR cost effective* OR cost utility OR cost-utility OR cost benefit OR cost-benefit OR quality adjusted life years OR health economic OR economic evaluation
Economic evaluation model related terms	6	Markov model OR discrete event stimulation OR decision tree OR decision-analytic model OR decision analytic model OR microsimulation OR micro-simulation OR agent-base* OR agent base*
	7	#3 and #4 and #5 and #6

Quality assessment of primary studies using ECOBIAS checklist

Table s4. Quality assessment of primary studies using ECOBIAS checklist

	Bias	Questions considered in evaluating for the risk of bias, as per ECOBIAS checklist[1]	How the gradings were offered
**PART A: Overall checklist for bias in economic evaluation**			
	Narrow perspective bias	• Was a societal perspective adopted?<br>• If not, has a different perspective been justified?	Studies that either used a societal perspective or justified their chosen perspective were graded as having a low risk of bias. Conversely, studies that did neither were graded as having a high risk of bias. Among the reviewed studies, only one study[2] used a societal perspective, while six reported a healthcare provider perspective[3-8] and the remainder reported a healthcare payer perspective. Only the economic evaluation by Ontario Health[9] provided a justification for the perspective used and was therefore graded as having a partial risk for the narrow perspective bias.
	Inefficient comparator bias	• Was the best alternative chosen as comparator?<br>• Was current practice chosen as a comparator? Have all comparators been described in sufficient detail?	The best alternative or current practice for stroke prophylaxis was determined as the therapeutic modalities most widely used within the relevant jurisdiction, a practice well-established in guidelines governing economic evaluations [10, 11]. Consequently, studies that compared LAAC with all commonly used oral anticoagulants (warfarin, dabigatran, apixaban, and rivaroxaban) for a base case population without contraindications for oral anticoagulants were graded as having a low risk for this bias. All the studies provided sufficient detail in describing the chosen comparator.
	Cost-measurement omission bias	• Were all costs relevant to the disease and intervention identified and considered?	None of the studies included implementation costs for LAAC or oral drugs, a significant category of costs, especially for LAAC, which requires specialized staff, equipment, and space. Consequently, all studies were graded as having a high risk for this bias.<br><br>All studies except one [8] presented the items/events they costed with relevant references in a table. However, some studies were not exhaustive in listing costs. For example, four studies[5, 12-14] did not detail how peri-procedural complications of the LAAC procedure were costed, despite including these complications in the model structure. Additionally, some studies[5, 6, 8, 12-15] did not include follow-up care for LAAC, which is a significant omission.
	Intermittent data collection bias	• Was the resource use measured continuously?	The checklist emphasizes the importance of having insight into all resource use (costs) throughout the entire follow-up period[16]. Previous studies suggested estimating total costs based on intermittently collected data, such as three months within a one-year period[17, 18], but it is now known to lead to bias[19].<br>Studies that accounted for follow-up care costs did not report whether the data were collected continuously or intermittently, making them vulnerable to receiving a high-risk grading due to inadequate reporting.
	Invalid valuation bias	• Is the price calculation presented in a detailed manner?<br>• Have reference prices been used?	Studies often included references for the costs used in the economic model, but they did not provide detailed calculations. We graded studies as having a low risk for this bias if they included valid references, such as reference prices and costs from previous literature, as recommended[1].<br>Two studies received a partial risk grading for this bias. One study[3] did not use local reference prices when available, and another study[8] did not provide references for all the cost items used in the model.

	Ordinal ICER bias	• Have cardinal scales for the outcomes measure in a CEA been used?	All authors measured health outcomes in atrial fibrillation using QALY, which employs a cardinal scale. This approach does not account for the ordinal ICER bias.
	Double counting bias	• Are variables adequately checked for double counting?	Double-counting bias can occur in economic evaluation when a parameter is counted more than once. In cost-utility analyses, it can happen when the consequences of a treatment are included in both the cost side (numerator) and the estimation of quality weights, i.e., QALY (denominator)[16].<br>While none of the studies reported checking for double-counting bias, our examination of the costs and QALYs incorporated showed that none of the studies had a high risk for this bias.
	Inappropriate discounting bias	• Have discounting rates from guidelines been applied?	Most authors in the primary analyses referenced local guidelines for the discount rate used in their models[2, 4-8, 13, 14]. Studies were considered to have a high-risk bias when they did not provide the discount rate or reference for the discount rate used.
	Limited sensitivity analysis bias	• Have the four principles of uncertainty (methodological, structural, heterogeneity, parameter) been considered in sufficient detail?	All studies received a high-risk bias rating for sensitivity analyses, as they were limited to addressing parameter uncertainty and lacked adequate reporting.
	Sponsor bias	• Have sponsorships been disclosed?<br>• Is the study protocol freely accessible?	Most studies[3-6, 12-15] disclosed sponsorships and conflicts of interest. The study without funding information[9] received a high-risk bias rating due to inadequate reporting. The remaining studies received a partial risk rating for this bias because they did not provide freely accessible links to their study protocols. While this protocol accessibility question may not fully apply to model-based economic evaluations, we followed the methodology for these ratings as described.
	Reporting and dissemination bias	• Has the study/trial been listed in a trial register?<br>• Have all results been reported according to the study protocol?	None of the studies were registered in a trial register. However, we believe that this question may not be entirely applicable to model-based economic evaluations. Due to the absence of trial registration, we could not assess whether the results were reported according to the study protocol. As a result, all the studies were rated as having a high-risk bias due to inadequate reporting.
**PART B: Model-specific aspects of bias in economic evaluation**			
	Structural assumption bias	• Is the model structure in line with coherent theory?<br>• Do treatment pathways reflect the nature of disease?	None of the studies specified the sources of data used to develop the model structure. While the structure of the model was consistent with the condition being investigated in the majority of studies, one study[3] excluded the state of myocardial infarction, citing unavailability of input data. It's important to note that good practice guidelines recommend that the conceptual structure of a model should be driven by the decision problem, not by data availability[20]. Therefore, this study was marked as having a high risk for this bias.<br>In another study[2] the health states only contained ischaemic stroke, categorized according to the modified Rankin scale. None of the other common complications were included in the model structure, leading to a high-risk grading for this bias.<br><br>Additionally, an important aspect of data identification is adherence to treatment. Compliance has been shown to significantly influence ICER[21], Therefore, it is recommended that accurate information about adherence be incorporated into the model[1]. Poor compliance is a significant problem among AF patients[22], but only four studies (41,42,44,48incorporated treatment adherence rates into the model. Although this could potentially impact the final reported ICER, we did not take this into account when grading the studies.
	No treatment comparator bias	• Is there an adequate comparator, i.e. care as usual?	All studies made appropriate comparisons aligned with their research questions, resulting in a low risk of no treatment comparator bias.
	Wrong model bias	• Is the model chosen adequate regarding the decision problem?	All studies utilised Markov models, an appropriate choice given the chronic nature of AF, where recurring events such as stroke and bleeding occur.
	Limited time horizon bias	• Was a lifetime horizon chosen?<br>• Were shorter time horizons	Considering a lifetime time horizon seems important in AF as the risk of disease recurrence and complications increase with age. Majority (11/12) used a 20-year time horizon, without explicitly labeling it as 'lifetime,' which is appropriate given the increasing

		adequately justified?	risk of disease recurrence and complications with age in AF. However, one study[3] adopted a 10-year horizon, starting with a cohort aged 65 years, potentially insufficient to capture all relevant costs and outcomes. This shorter horizon lacked justification, leading to a high-risk bias rating.
	Bias related to data identification	• Are the methods of data identification transparent?<br>• Are all choices justified adequately?<br>• Do the input parameters come from high quality and well-designed studies?	Among the reviewed studies, only one study[2] provided information on how data sources were identified, and none of the studies provided justifications for their choice of data sources. It's worth noting that the determination of data source quality can be subjective. However, concerns were raised regarding the methodological quality of the PROTECT-AF and PREVAIL trials[23-25] when these trials were exclusively used as sources for clinical parameters in LAAC.<br>The majority of studies received a high-risk rating for inadequate reporting because they could not satisfactorily address two or more of the three key questions outlined in the left column of the assessment tool.
	Bias related to baseline data	• Are probabilities, for example, based on natural history data?<br>• Is transformation of rates into transition probabilities done accurately?	All studies, except for Labori and colleagues[2] extensively utilized data from the PROTECT-AF and/or PREVAIL trials, which compared LAAC with warfarin. Labori and colleagues chose not to use data from these trials due to differences in the study populations.<br>Similar to the approach taken by Labori et al., many other studies also encountered disparities between the characteristics of the model population and those of the trial population, such as differences in contraindications to oral anticoagulants (OAC), mean stroke risk, and bleeding risk. Despite these discrepancies, they relied on trial data for model inputs.<br>Additionally, none of the studies provided adequate descriptions of how the rates from the trials were transformed into transition probabilities. This lack of detail made them susceptible to receiving a high-risk rating due to inadequate reporting.
	Bias related to treatment effects	• Are relative treatment effects synthesized using appropriate metanalytic techniques?<br>• Are extrapolations documented and well justified?<br>• Are alternative assumptions explored regarding extrapolation?	Studies that compared LAAC with novel oral anticoagulants (OAC) employed various indirect comparison techniques to derive treatment effects. Most studies converted treatment effects for LAAC and warfarin from pivotal trials, with the exception of one study[9] which conducted a meta-analysis utilizing recent data extracted from trials, observational studies, and registries[26].<br>Notably, some studies [5, 8, 15] opted to use only PROTECT-AF trial data to derive clinical inputs for LAAC, excluding PREVAIL trial data. This choice may have introduced bias, as the PREVAIL trial failed to demonstrate the non-inferiority of LAAC against warfarin for ischaemic stroke prevention [27].<br>Unfortunately, none of the studies provided detailed justifications for their extrapolations or explored alternative assumptions regarding extrapolations. Consequently, all studies received a high-risk rating due to inadequate reporting.
	Bias related to quality-of-life weights (utilities)	• Are the utilities incorporated appropriate for the specific decision problem?	Some studies provided only the mean utility score used for model input parameters[3, 4], while others offered brief descriptions of the methods used to estimate this mean score[5, 12-14]. A few studies mentioned using quality-of-life short form-12 data collected during the Watchman ASAP trial[5] and PROTECT-AF trial [13, 14] to derive QALY estimates for LAAC. In contrast, three studies[6-8] reported a lack of quality-of-life estimates for LAAC and instead utilized relevant data from percutaneous coronary interventions.<br>However, despite differences in data sources, all studies received a low-risk rating for this bias, as the utilities incorporated were deemed appropriate for the specific decision problem.
	No transparent data incorporation bias	• Is the process of data incorporation transparent?<br>• Are all data and their sources described in detail?	All studies comparing LAAC with a novel OAC, except for the study by Labori and colleagues which employed a meta-analysis, utilized indirect comparison techniques to derive clinical inputs for LAAC. This was because the available pivotal trials had compared LAAC only with warfarin. However, only one study[9] provided a clear presentation of how this indirect comparison was conducted.<br>Moreover, although three out of ten studies[5, 7, 8] reported using patient-level Markov microsimulation models, none of them specified transition probabilities for the events listed in their tables based on patient-level data. As a result, these studies were assigned a high-risk bias rating due to inadequate reporting.
	Limited scope bias	• Have the four principles of uncertainty (methodological, structural, heterogeneity, parameter) been considered?	All included studies presented and discussed probabilistic sensitivity analyses, which are typically employed to evaluate parameter uncertainty. However, they did not address methodological or structural uncertainty in their analyses. This omission exposed the studies to a high risk of limited scope bias due to inadequate reporting.
	Bias related to internal consistency.	• Has internal consistency in terms of mathematical logic been evaluated?	None of the primary studies reported an analysis for the internal consistency of the model, as recommended by best practice guidelines[1].

1. Adarkwah CC, van Gils PF, Hiligsmann M, Evers SMAA. Risk of bias in model-based economic evaluations: the ECOBIAS checklist. *Expert Review of Pharmacoeconomics & Outcomes Research*. 2016/07/03 2016;16(4):513-523. doi:10.1586/14737167.2015.1103185
2. Labori F, Persson J, Bonander C, Jood K, Svensson M. Cost-effectiveness analysis of left atrial appendage occlusion in patients with atrial fibrillation and contraindication to oral anticoagulation. *Eur Heart J*. Dec 17 2021;doi:10.1093/eurheartj/ehab847
3. Kawakami H, Nolan MT, Phillips K, Scuffham PA, Marwick TH. Cost-effectiveness of combined catheter ablation and left atrial appendage closure for symptomatic atrial fibrillation in patients with high stroke and bleeding risk. *Am Heart J*. Jan 2021;231:110-120. doi:10.1016/j.ahj.2020.08.008
4. Lee VW, Tsai RB, Chow IH, et al. Cost-effectiveness analysis of left atrial appendage occlusion compared with pharmacological strategies for stroke prevention in atrial fibrillation. *BMC Cardiovasc Disord*. Aug 31 2016;16(1):167. doi:10.1186/s12872-016-0351-y
5. Reddy VY, Akehurst RL, Armstrong SO, et al. Cost effectiveness of left atrial appendage closure with the Watchman device for atrial fibrillation patients with absolute contraindications to warfarin. *Europace*. Jul 2016;18(7):979-86. doi:10.1093/europace/euv412
6. Saw J, Bennell MC, Singh SM, Wijeysundera HC. Cost-Effectiveness of Left Atrial Appendage Closure for Stroke Prevention in Atrial Fibrillation Patients With Contraindications to Anticoagulation. *Can J Cardiol*. Nov 2016;32(11):1355.e9-1355.e14. doi:10.1016/j.cjca.2016.02.056
7. Singh SM, Micieli A, Wijeysundera HC. Economic evaluation of percutaneous left atrial appendage occlusion, dabigatran, and warfarin for stroke prevention in patients with nonvalvular atrial fibrillation. *Circulation*. Jun 18 2013;127(24):2414-23. doi:10.1161/circulationaha.112.000920
8. Micieli A, Wijeysundera HC, Qiu F, Atzema CL, Singh SM. A Decision Analysis of Percutaneous Left Atrial Appendage Occlusion Relative to Novel and Traditional Oral Anticoagulation for Stroke Prevention in Patients with New-Onset Atrial Fibrillation. *Med Decis Making*. Apr 2016;36(3):366-74. doi:10.1177/0272989x15593083
9. Health Quality Ontario. Left Atrial Appendage Closure Device With Delivery System: A Health Technology Assessment. *Ont Health Technol Assess Ser*. 2017;17(9):1-106.
10. Drummond M, Sculpher M. Common methodological flaws in economic evaluations. *Med Care*. Jul 2005;43(7 Suppl):5-14. doi:10.1097/01.mlr.0000170001.10393.b7
11. Sculpher MJ, Claxton K, Drummond M, McCabe C. Whither trial-based economic evaluation for health care decision making? *Health Econ*. Jul 2006;15(7):677-87. doi:10.1002/hec.1093
12. Reddy VY, Akehurst RL, Amorosi SL, Gavaghan MB, Hertz DS, Holmes DR, Jr. Cost-Effectiveness of Left Atrial Appendage Closure With the WATCHMAN Device Compared With Warfarin or Non-Vitamin K Antagonist Oral Anticoagulants for Secondary Prevention in Nonvalvular Atrial Fibrillation. *Stroke*. Jun 2018;49(6):1464-1470. doi:10.1161/strokeaha.117.018825
13. Reddy VY, Akehurst RL, Armstrong SO, Amorosi SL, Beard SM, Holmes DR, Jr. Time to Cost-Effectiveness Following Stroke Reduction Strategies in AF: Warfarin Versus NOACs Versus LAA Closure. *J Am Coll Cardiol*. Dec 22 2015;66(24):2728-2739. doi:10.1016/j.jacc.2015.09.084
14. Reddy VY, Akehurst RL, Gavaghan MB, Amorosi SL, Holmes DR, Jr. Cost-Effectiveness of Left Atrial Appendage Closure for Stroke Reduction in Atrial Fibrillation: Analysis of Pooled, 5-Year, Long-Term Data. *J Am Heart Assoc*. Jul 2 2019;8(13):e011577. doi:10.1161/jaha.118.011577

15. Freeman JV, Hutton DW, Barnes GD, et al. Cost-Effectiveness of Percutaneous Closure of the Left Atrial Appendage in Atrial Fibrillation Based on Results From PROTECT AF Versus PREVAIL. *Circ Arrhythm Electrophysiol*. Jun 2016;9(6)doi:10.1161/circep.115.003407
16. Evers SM, Hiligsmann M, Adarkwah CC. Risk of bias in trial-based economic evaluations: identification of sources and bias-reducing strategies. *Psychol Health*. Jan 2015;30(1):52-71. doi:10.1080/08870446.2014.953532
17. Goossens MEJB, Mölken MPMHR-v, Vlaeyen JWS, van der Linden SMJP. The cost diary: a method to measure direct and indirect costs in cost-effectiveness research. *Journal of Clinical Epidemiology*. 2000/07/01/ 2000;53(7):688-695. doi:https://doi.org/10.1016/S0895-4356(99)00177-8
18. Lamoureux EL, Chou SL, Larizza MF, Keeffe JE. The Reliability of Data Collection Periods of Personal Costs Associated with Vision Impairment. *Ophthalmic Epidemiology*. 2006/01/01 2006;13(2):121-126. doi:10.1080/09286580500536781
19. Hendriks MRC, Al MJ, Bleijlevens MHC, et al. Continuous versus Intermittent Data Collection of Health Care Utilization. *Medical Decision Making*. 2013;33(8):998-1008. doi:10.1177/0272989x13482045
20. Roberts M, Russell LB, Paltiel AD, Chambers M, McEwan P, Krahn M. Conceptualizing a model: a report of the ISPOR-SMDM Modeling Good Research Practices Task Force--2. *Value Health*. Sep-Oct 2012;15(6):804-11. doi:10.1016/j.jval.2012.06.016
21. Hiligsmann M, Boonen A, Rabenda V, Reginster JY. The importance of integrating medication adherence into pharmacoeconomic analyses: the example of osteoporosis. *Expert Rev Pharmacoecon Outcomes Res*. Apr 2012;12(2):159-66. doi:10.1586/erp.12.8
22. Ozaki AF, Choi AS, Le QT, et al. Real-World Adherence and Persistence to Direct Oral Anticoagulants in Patients With Atrial Fibrillation: A Systematic Review and Meta-Analysis. *Circ Cardiovasc Qual Outcomes*. Mar 2020;13(3):e005969. doi:10.1161/circoutcomes.119.005969
23. Sohaib SM, Fox KF. A meta-analysis of left atrial appendage closure for stroke prevention in atrial fibrillation-adding to the debate but elements remain unresolved. *J Thorac Dis*. Aug 2015;7(8):E226-9. doi:10.3978/j.issn.2072-1439.2015.08.03
24. Ferlini M, Rossini R. Left atrial appendage closure: Six reasons why I wouldn't choose a percutaneous closure for my appendage. *International Journal of Cardiology*. 2018/01/15/ 2018;251:42-44. doi:https://doi.org/10.1016/j.ijcard.2017.09.015
25. Mandrola J, Foy A, Naccarelli G. Percutaneous left atrial appendage closure is not ready for routine clinical use. *Heart Rhythm*. Feb 2018;15(2):298-301. doi:10.1016/j.hrthm.2017.10.007
26. Labori F, Bonander C, Persson J, Svensson M. Clinical follow-up of left atrial appendage occlusion in patients with atrial fibrillation ineligible of oral anticoagulation treatment-a systematic review and meta-analysis. *J Interv Card Electrophysiol*. Aug 2021;61(2):215-225. doi:10.1007/s10840-021-00953-9
27. Pokorney SD, Mark DB. Cost-Effectiveness of Left Atrial Appendage Occlusion. *Circulation: Arrhythmia and Electrophysiology*. 2016;9(6):e004223. doi:doi:10.1161/CIRCEP.116.004223
